# Two-month ketogenic diet alters systemic and brain metabolism in middle-aged female mice

**DOI:** 10.1007/s11357-024-01314-w

**Published:** 2024-08-24

**Authors:** Kirsten J. Roslund, Jon J. Ramsey, Jennifer M. Rutkowsky, Zeyu Zhou, Carolyn M. Slupsky

**Affiliations:** 1 Department of Nutrition, University of California Davis, Davis, CA USA; 2 Department of Molecular Biosciences, School of Veterinary Medicine, University of California Davis, Davis, CA USA; 3 Department of Food Science & Technology, University of California Davis, Davis, CA USA

**Keywords:** Ketogenic diet, Ketones, Metabolism, β-Hydroxybutyrate, Aging, Female

## Abstract

The ketogenic diet (KD) is a very low-carbohydrate, high-fat diet that reduces glucose catabolism and enhances β-oxidation and ketogenesis. While research in female rodents is limited, research in male rodents suggests that ketogenic interventions initiated at midlife may slow age-related cognitive decline, as well as preserve muscle mass and physical function later in life. This study aimed to investigate the effects of a KD on global metabolic changes in middle-aged females to inform potential mechanisms behind the anti-aging effects of this diet in an understudied sex. Targeted ^1^H-NMR metabolomics was conducted on serum, the liver, the kidney, and the gastrocnemius muscle, as well as the cortex and the hippocampal brain regions in 16-month-old female mice after a 2-month KD. Analysis of the serum and liver metabolome revealed that the 2-month KD resulted in increased concentrations of fatty acid catabolism metabolites, as well as system-wide elevations in ketones, consistent with the ketogenic phenotype. Metabolites involved in the glucose-alanine cycle were altered in the gastrocnemius muscle, serum and the liver. Other tissue-specific alterations were detected, including distinct effects on hepatic and renal one-carbon metabolism, as well as region specific differences in metabolism across hippocampal and cortical parts of the brain. Alterations to hippocampal metabolites involved in myelinogenesis could relate to the potential beneficial effects of a KD on memory.

**Supplementary Information:**

The online version contains supplementary material available at 10.1007/s11357-024-01314-w.

## Introduction

The ketogenic diet is a high-fat, moderate-protein, carbohydrate-restricted eating pattern [1]. It was duly named ketogenic, as it stimulates the process of ketogenesis, the synthesis of ketone bodies acetoacetate, β-hydroxybutyrate, and acetone [2]. Similar to prolonged fasting, initiation of a very low carbohydrate diet lowers insulin concentrations, driving the release of fatty acids from adipose tissue into circulation [3]. Once in the liver, fatty acids are transported to the mitochondria and catabolized through β-oxidation into acetyl-CoA, which may enter the citric acid cycle or be diverted to ketogenesis [3]. Acetoacetate and β-hydroxybutyrate are essential energy metabolites for the brain during glucose depletion, while also supporting the energy needs of other organs including skeletal muscle, heart, renal cortex, and the intestinal mucosa [4].

The ketogenic diet was initially developed as an alternative to fasting for the treatment of intractable epilepsy [1]; however, it is now gaining popularity as a method for weight loss [5], as well as for improving cognition [6] and physical function [7]. Indeed, several studies in mouse models have demonstrated that a ketogenic diet initiated at middle-age improved lifespan [7, 8], as well as other aspects of aging, including age-related memory loss [7, 8], sarcopenia [9], and physical function [7]. Aging is accompanied by many biological changes, including alterations in cellular metabolism that may be a result of and/or contribute to diminished cognitive and physical function with time. Thus, investigations into the system-wide metabolic changes induced by a ketogenic diet at midlife could provide insight into the anti-aging aspects of this diet, as well as provide potential mechanisms through which a ketogenic diet may benefit age-related outcomes.

Most studies investigating the metabolomic phenotype of a ketogenic diet have focused on male mice. Recent studies have demonstrated sex-specific differences in response to a short-term KD in adult C57BL/6 mice, including impacts on body weight, and adiposity, as well as cardiac and skeletal muscle mass [10, 11]. Given this divergence in response, investigations into the impact of a KD on global metabolism in female mice is warranted. Thus, this study aimed to fill critical gaps in the literature through investigation into the systemic metabolic effects of a 2-month KD on middle-aged female mice through analysis of the serum metabolome and the tissue metabolomes of major metabolic organs including the liver, kidney, and gastrocnemius muscle, as well as the cortex and hippocampal regions of the brain.

## Methods

### Animals

Female C57BL/6JN mice (*n* = 16) used in this study were a subset of mice from a larger study investigating the effects of a ketogenic diet on cognitive function, motor function, and biochemical changes in several tissues, and a more detailed overview of the study design has been previously reported [12]. Briefly, mice were obtained from the NIA Aged Rodent Colony at 12 months of age, group-housed in a temperature (22–24 °C) and humidity (40–60%) controlled room and maintained on a 12-h light/dark cycle. Daily health checks were conducted. All animal protocols were approved by the UC Davis Institutional Animal Care and Use Committee and were in accordance with the NIH guidelines for the Care and Use of Laboratory Animals.

### Dietary interventions

Upon arrival, mice were provided ad libitum chow (LabDiet 5001). At 14 months of age, mice were individually housed and counterbalanced by body weight to a control (CD; *n* = 8), or ketogenic diet (KD; *n* = 8) provided at isocaloric amounts (11.2 kcal/day). Semi purified diets were prepared on site. CD mice were provided a modified AIN-93G diet [13], altered to match the lower protein content of the KD. Full compositional details are provided in Table 1. The CD diet was comprised of (% of total kcal) 10% protein, 74% carbohydrate, and 16% fat. The KD contained 10% protein, < 0.5% carbohydrate, and 89.5% fat. Due to the carbohydrate carriers present in the CD mineral mix TD.94046, the KD was formulated with mineral mix TD.98057.

**Table 1:** Composition of the experimental diets

	Control	Ketogenic
Energy density (kcal/g)	3.8	6.7
kcal/day	11.2	11.2
Ingredients	g/kg diet	
Casein	111.0	191.0
dl-methionine	1.5	2.7
Corn starch	490.0	0.0
Maltodextrin	132.0	0.0
Sucrose	100.0	0.0
Mineral mix TD98057 (has sucrose)	35.0	24.0
Vitamin mix CA40060 (has corn starch)	10.0	18.0
Soybean oil	70.0	70.0
Lard	0	582
Calcium phosphate dibasic	0	19.3
Calcium carbonate	0	8.2
Cellulose	48.0	85.0
Potassium phosphate monobasic	2.4	0
TBHQ	0.014	0.126

### Tissue and blood collection

After 2 months on diet (16 months of age), all mice were fasted overnight (12 h) and then anesthetized through inhalation of isoflurane (5%). Blood was collected through cardiac puncture, followed by tissue collection. The gastrocnemius, liver, kidney, hippocampus, and cortex were flash frozen in liquid nitrogen. Samples were stored at − 80 °C until preparation for metabolomics analyses. Blood was transported on ice and left to sit at room temperature between 30 and 60 min. Blood samples were centrifuged (15 min, 1600* g*, 4 °C) and serum was removed and stored at − 80 °C until filtration.

### Serum filtration

Serum samples were thawed on ice. Amicon Ultra-0.5 mL 3000 molecular weight centrifugal filters (Millipore, Burlington, MA, USA) were washed three times with Mili-Q® ultrapure water to remove glycerol and centrifuged (5 min, 22 °C, 14 krcf) after each wash. A maximum of 350 μL serum was transferred onto dried Amicon filters and centrifuged (60 min, 4 °C, 14 krcf) to remove proteins and lipids. Filtered serum samples were stored at − 80 °C for at least 12 h, and subsequently dried using evaporative centrifugation until completely dry followed by storage at − 80 °C until reconstitution for metabolomics analysis.

### Tissue sample preparation

Tissues were separately cryoground in liquid nitrogen and stored at − 80 °C until extraction. To obtain polar metabolites, the polar layer was isolated using a modified Folch extraction following methods outlined by Hasegawa et al. [14]. Briefly, frozen cryoground samples were weighed and 2.4 mL of 2:1 chloroform:methanol solvent with 0.002% butylated hydroxytoluene was added per 30 mg of tissue. Subsequently, 600 μL of 1 mM ethylenediamine tetraacetic acid with KCl (9 g/L) was added, and samples were thoroughly mixed using a vortexer and centrifuged (15 min, 2000 rpm, 0 °C). The nonpolar layer (bottom) was removed and discarded. To the remaining sample, 1.4 mL of a 10:1 chloroform:methanol solvent was added, and samples were thoroughly mixed using a vortexer and centrifuged (15 min, 2000 rpm, 0 °C). The polar (top) layer was carefully aspirated using a pipette and centrifuged (2 min, 10 krcf, room temperature). To produce comparable drying times, Mili-Q® ultrapure water was added to ensure all samples had equivalent volumes. Samples were dried to completion using evaporative centrifugation and stored at − 80 °C until preparation for metabolomics analysis.

### Metabolomics

Dried tissue and serum samples were reconstituted in 270 μL of 100 mM phosphate-buffered solution prepared in D_2_O and vortexed until dissolved. Samples were centrifuged (1 min, 2000 rpm), transferred to a new tube, and centrifuged again (15 min, 4 °C, 14 krcf). A total of 207 μL of the reconstituted sample was added to a new tube followed by the addition of 23 μL of the internal standard 5.0 mM 3-(trimethylsilyl)-1-propanesulfonic acid-d_6_ dissolved in 99.9% v/v D_2_O and 0.1% w/v NaN_3_ (Chenomx, Edmonton, AB, USA). The pH of the sample was adjusted to pH 6.8 ± 0.1 and 180 μL of each sample was loaded into 3-mm NMR tubes (Bruker, Billerica, MA, USA). Targeted metabolomics analysis was conducted using ^1^H-NMR spectroscopy on a Bruker Avance 600 MHz spectrometer (Bruker, Billerica, MA, USA) at the UC Davis NMR Facility. As described previously [15], ^1^H-NMR spectra were acquired using the noesypr1d pulse sequence with the modification of 128 transients to improve the signal-to-noise ratio. Metabolite identification and quantification was completed using a 600-MHz compound library in Chenomx NMR Suite (version 8.6, Chenomx, Edmonton, AB, USA). Absolute quantification of each metabolite was determined based on the known volume of the internal standard added (23 μL) to each sample (207 μL), for a total volume of 230 μL. Each sample was reconstituted with 270 μL phosphate buffer in D_2_O. Serum metabolite concentrations were corrected as follows:

$${\left[Metabolite\right]}_{f}={\left[Metabolite\right]}_{Chenomx}\times \frac{270\mu L\left(\frac{230\mu L}{207\mu L}\right)}{Vo{l}_{filtrate}},$$

where units are expressed as μM.

Tissue metabolite concentrations were calculated as follows:

$${\left[Metabolite\right]}_{f}={\left[Metabolite\right]}_{Chenomx}\times \frac{\left(\frac{Vo{l}_{estimated}}{Vo{l}_{collected}}\right)\times 270\mu L\times \left(\frac{230\mu L}{207\mu L}\right)}{Mas{s}_{tissue}},$$

where vol_estimated_ is the estimated total volume of the polar layer, vol_collected_ is the volume of polar layer collected, and units are expressed as nmol/g.

### Statistical analyses

Statistical analyses were completed using R (version 4.0.4) and RStudio (version 2022.02.2 Build 492). All 16 female mice that completed the 2-month intervention were included in the data set (CD = 8, KD = 8), with the following exceptions. One kidney sample from the CD group was lost due to a sample preparation error and one hippocampal sample from the CD group was too small (< 4 mg) to accurately quantify all metabolites and, thus, both were excluded from analyses. Due to several orders of magnitude difference between raw concentrations of metabolites, sample concentrations were log_10_-transformed as a method of scaling the data to reduce the weight of this difference for multivariate analyses. Non-metric multi-dimensional scaling (NMDS) was performed to visualize the metabolome in two dimensions. The fewest number of dimensions were selected for each NMDS plot that also maintained a stress score less than 0.05. To assess if there was an effect of diet on the metabolome, the homogeneity of group variances (beta-dispersion) was tested, followed by permutational multivariate analysis of variance (PERMANOVA). Significance was defined as *p* < 0.05 for PERMANOVA. For serum and all tissues except the gastrocnemius, metabolite concentrations were not normally distributed following log_10_-transformation. All quantified metabolites in the gastrocnemius, except for two that could not be normalized with transformation, were log_10_-transformed and Welch’s *t*-tests were conducted to determine univariate effects of diet on each metabolite (mean ± standard deviation). Wilcoxon rank-sum test was performed on all untransformed concentrations to assess univariate effects of diet on each metabolite (median and interquartile range). Univariate effect sizes were calculated using Hedge’s g (*g*). Due to the small sample size, differences between CD and KD groups were defined as statistically significant by an unadjusted Wilcoxon rank sum test or Welch’s *t*-test result of *p* < 0.05 and at least a large Hedge’s *g* effect size |*g*|≥ 0.8; a very large effect size is indicated by |*g*|≥ 1.2. A statistical trend for a difference was defined as an unadjusted Wilcoxon rank sum test or Welch’s *t*-test result of *p* < 0.1 and a Hedge’s *g* effect size |*g*|≥ 0.8. To investigate the potential associations between metabolites of interest, Spearman correlation tests were conducted to determine associations overall and by group.

## Results

In this study, we sought to assess the effects of a 2-month ketogenic diet (KD) on the serum and tissue metabolomes in middle-aged (16-month-old) female mice using ^1^H-NMR to identify and quantify water-soluble metabolites in the serum, liver, kidney, gastrocnemius muscle, and brain tissue. We previously published that the KD had no significant impact on female body weight; however, percent body fat mass trended higher [12].

### Effect of the KD on serum metabolism

**Fig. 1:**
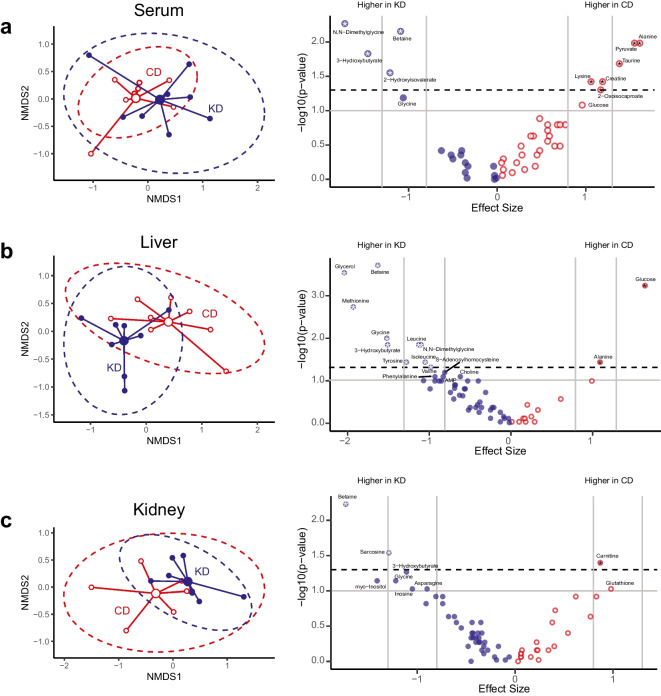
Impact of a 2-month ketogenic diet on the serum, liver, and kidney metabolomes. Non-metric multidimensional scaling (NMDS) plots (left) of log transformed data with 95% confidence ellipses and corresponding metabolite Volcano plots of Hedge’s g effect sizes versus log-transformed *p*-values (right) in **a** serum (NMDS *p* = 0.027, stress = 0.040), **b** liver (NMDS *p* = 0.0049, stress = 0.030), and **c** kidney (NMDS *p* = 0.075, stress = 0.035). Mice in the control group (CD) are indicated in red, and mice in the ketogenic diet group are indicated in blue. In the volcano plots, metabolites higher than the horizontal dotted line have *p*-values < 0.05, and those above the horizontal grey line have *p*-values < 0.1. The vertical grey lines represent Hedge’s *g* (*g*) cutoffs for large (|*g*|= 0.8) and very large (|*g*|= 1.2) effect sizes. A negative effect size indicates that the metabolites are larger in mice on the ketogenic diet (KD, blue) whereas a positive effect size indicates metabolites are larger in mice on the control diet (CD, red). Metabolites with an asterisk are considered significant (large or very large effect size and *p*-value < 0.05). Metabolites trending toward significance (large or very large effect size and *p*-value < 0.1) are also indicated

NMDS was performed on quantified serum metabolites, which revealed a significant difference between dietary groups (PERMANOVA; *R*^2^ = 0.13, *p* = 0.027 under 9999 permutations, Fig. 1a). When fasting or consuming a KD, the body enters a state of nutritional ketosis, a metabolic state characterized by increased fatty acid catabolism and ketone body production in response to reduced glucose availability. Several metabolites were either significantly different or trended towards different between mice on the KD vs. CD (Fig. 1a), which are summarized in Table 2. Significantly higher β-hydroxybutyrate (βHB), the most abundant ketone, was observed in the KD group compared to the CD group, which is consistent with results from middle-aged C57BL/6 male mice after 1 month on a KD [3]. A trend for lower serum glucose in KD compared to CD mice was also observed. Higher concentrations of 2-hydroxyisovalerate, a metabolite that is a product of valine metabolism [16], as well as metabolites involved in one-carbon metabolism (dimethylglycine, betaine), were either significantly or trending towards significantly higher in the KD group compared to the CD group. In the KD group, lower concentrations of glucose-alanine cycle intermediates, alanine and pyruvate, were observed. Other serum metabolites that were lower in mice on the KD included the bile acid conjugate taurine, the phosphate carrier creatine, the leucine catabolite 2-oxoisocaproate, and the essential amino acid lysine.

**Table 2:** Metabolites significantly different or trending toward a difference between mice on either the control (CD) or ketogenic (KD) diet in serum (expressed as μM) and liver (expressed as nmol/g). For each metabolite, median and interquartile range (IQR), Wilcoxon-rank sum *p*-value (*p*), and Hedge’s g effect size (*g*) are indicated

Metabolite	Serum				Liver			
	CD, median (IQR)	KD, median (IQR)	*p*	*g*	CD, median (IQR)	KD, median (IQR)	*p*	*g*
2-Hydroxyisovalerate	6 (5.3–6.5)	7.7 (6.4–8.5)	0.0281*	− 1.21	-	-	-	-
2-Oxoisocaproate	5.1 (3.3–6.7)	3 (2.1–3.7)	0.0499*	1.17	-	-	-	-
β-Hydroxybutyrate	867 (527.4–1391)	2006.3 (1372.1–2538.3)	0.0148*	− 1.46	508.9 (331.6–837.1)	1129.8 (807.6–1373.5)	0.0148*	− 1.50
Alanine	466.5 (391.1–476)	286 (225–358.9)	0.0104*	1.61	2680.2 (2281–2902.5)	2150.4 (2008.4–2434.3)	0.0379*	1.10
AMP	-	-	-	-	591.4 (530.7–756.3)	739.2 (719.3–842.2)	0.0830^†^	− 0.82
Betaine	58 (53.9–67.7)	87 (82.6–99.9)	0.0070*	− 1.09	326.9 (261.8–389.4)	948.3 (856.6–1250.9)	0.0002*	− 1.62
Creatine	149 (141–168.9)	118 (104.6–133.7)	0.0379*	1.19	-	-	-	-
Glucose	10,144.6 (9892.5–10,899.2)	7808.6 (7374.1–9710.1)	0.0830^†^	0.96	39,599.2 (37,123.6–41,866.3)	32,085.5 (27,953.6–33,430.4)	0.0006*	1.65
Glycerol	-	-	-	-	185.5 (162.9–201.6)	308.4 (272.4–364.7)	0.0003*	− 2.03
Glycine	223.3 (204.5–237)	273 (227.1–315.1)	0.0650^†^	-1.06	1668.4 (1520.4–1898.2)	2236.3 (1951.5–2374.2)	0.0104*	− 1.51
Isoleucine	-	-	-	-	140.3 (126.1–151.8)	172.9 (151.7–204.3)	0.0379*	− 1.04
Leucine	-	-	-	-	242.1 (210.9–283.7)	324.4 (269–369.4)	0.0148*	− 1.12
Lysine	211.7 (201.4–236.1)	188.4 (166.7–206.1)	0.0379*	1.06	-	-	-	-
Methionine	-	-	-	-	29.9 (28.5–34.2)	46.1 (40.8–50.7)	0.0019*	− 1.92
Dimethylglycine	4.9 (4.6–5.4)	9.9 (8.5–10.7)	0.0054*	− 1.72	18 (16.8–20.6)	32.7 (26.8–39.4)	0.0148*	− 1.10
Phenylalanine	-	-	-	-	92 (86.1–99.7)	103.4 (97.1–119.8)	0.0830^†^	− 0.92
Pyruvate	109.8 (94.3–120.1)	68.2 (60.5–79.9)	0.0104*	1.55	-	-	-	-
S-Adenosylhomocysteine	-	-	-	-	26.4 (25.2–27.6)	28.5 (27.3–32.5)	0.0659^†^	− 0.80
Taurine	808.8 (683.3–874.6)	566.9 (463.4–655.9)	0.0207*	1.38	-	-	-	-
Tyrosine	-	-	-	-	129.7 (124.5–135.3)	161 (152.9–174.7)	0.0379*	− 1.27
Valine	-	-	-	-	224.2 (198.7–240.6)	260.1 (240.7–304.7)	0.0499*	− 0.97

*Metabolites significantly different between mice on the ketogenic diet vs. mice on the control diet (*p* < 0.05 and |g|≥ 0.8)

^†^Metabolites that trended toward a difference between mice on the ketogenic diet vs. mice on the control diet (*p* < 0.1 and |g|≥ 0.8)

-Metabolites not measured

All measured metabolite concentrations can be found in Supplementary Tables 1 and 2

### Effect of the KD on the liver metabolome

There was a significant difference by group in the liver metabolome (PERMANOVA; *R*^2^ = 0.16, *p* = 0.0049 under 9999 permutations, Fig. 1b, Table 2). In KD mice, higher βHB was observed in the liver, the major site of ketone production, whereas glucose was significantly lower. Glycerol, the gluconeogenic precursor derived from triglyceride breakdown, was also higher in the KD group, consistent with findings in 7-week-old male C57Bl/6 mice after an 8-week KD [17]. Similar to serum, the gluconeogenic amino acid alanine was significantly lower. Compounds involved in one-carbon metabolism including betaine, methionine, glycine, and dimethylglycine were significantly higher in the KD group, while S-adenosylhomocysteine trended higher. Branch-chained amino acids leucine, isoleucine, and valine were also significantly higher in the KD group. The aromatic amino acids tyrosine and phenylalanine were significantly higher and trending higher, respectively, in the KD group. Liver glycine and formate tended to be correlated overall (*p* = 0.0822, rho = 0.4500, Supplementary Fig. 1), with a significant positive correlation in the CD group (*p* = 0.0022, rho = 0.9286), but not the KD group (*p* = 0.8401, rho = 0.0952).

### Effect of the KD on the kidney metabolome

To determine if the kidney metabolome differed based on diet, NMDS was performed on quantified metabolites and PERMANOVA was used to assess differences in the centroids. Based on PERMANOVA, a statistical trend for a difference by group was observed for the kidney metabolome (*R*^2^ = 0.12, *p* = 0.075 under 9999 permutations, Fig. 1c, Table 3). βHB, an energy source in the renal cortex, tended to be higher in KD mice. The one-carbon metabolites betaine and sarcosine were significantly higher, while glycine and inosine trended higher, and glutathione trended lower in the KD group. Carnitine, the mitochondrial fatty acid transporter, was significantly lower in KD-fed mice. There was also a trend toward higher concentrations of the osmolyte myo-inositol.

**Table 3:** Metabolites significantly different or trending toward a difference between mice on either the control (CD) or ketogenic (KD) diet in kidney and gastrocnemius muscle (expressed as nmol/g). For each metabolite in the kidney, median and interquartile range (IQR), Wilcoxon-rank sum *p*-value (*p*), and Hedge’s g effect size (*g*) are indicated. For each metabolite in the gastrocnemius, log-transformed mean ± standard deviation (SD), p-value (*p*), and Hedge’s g effect size (*g*) are indicated

Metabolite	Kidney				Gastrocnemius			
	CD, Median (IQR)	KD, Median (IQR)	*p*	*g*	CD, Mean ± SD	KD, Mean ± SD	*p*	*g*
β-Hydroxybutyrate	305.5 (169.1–742.3)	898.3 (571.5–1166.9)	0.0541^†^	− 1.11	2.2 ± 0.33	2.63 ± 0.17	0.0072*	− 1.57
Alanine	1172.2 (967.7–1278)	1001.7 (764–1077.1)	0.2319	0.77	3.16 ± 0.09	3.06 ± 0.12	0.0882^†^	0.87
Asparagine	108.7 (87.1–126)	131.9 (121.3–148.1)	0.0939^†^	− 0.90	-	-	-	-
Betaine	1060.1 (990.6–1077.2)	1428.4 (1359.8–1801.1)	0.0059*	− 1.73	-	-	-	-
Carnitine	209 (197.5–244.5)	144.5 (124.1–181.3)	0.0401*	0.87	-	-	-	-
Glutamate	-	-	-	-	2.9 ± 0.06	3.03 ± 0.08	0.0020*	− 1.8
Glutathione^^^	271.7 (231.8–315.4)	221.3 (129.5–243.1)	0.0939^†^	0.98	287.1 (277.3–300.2)	310.5 (278.7–376.5)	0.2786	− 0.74
Glycine	2685.1 (2632.9–2881.9)	3395.1 (2894–3529.3)	0.0721^†^	− 1.22	3.24 ± 0.12	3.37 ± 0.12	0.0594^†^	− 0.97
Inosine	160.6 (130–189)	197.5 (180.9–240)	0.0939^†^	− 1.05	-	-	-	-
Myo-inositol	5673.3 (5259.4–5946.1)	7645.4 (5883.1–7992.5)	0.0721^†^	− 1.41	-	-	-	-
Sarcosine	6.2 (3.9–7.7)	9.5 (7.1–10.7)	0.0289*	− 1.29	-	-	-	-

*Metabolites significantly different between mice on the ketogenic diet vs. mice on the control diet (*p* < 0.05 and |*g*|≥ 0.8)

^†^Metabolites that trended toward a difference between mice on the ketogenic diet vs. mice on the control diet (*p* < 0.1 and |*g*|≥ 0.8)

-Metabolites not measured

^Normality could not be approximated using log transformation for glutathione in the gastrocnemius; median and interquartile range (IQR), Wilcoxon-rank sum *p*-value (*p*), and Hedge’s g effect size (*g*) are indicated

All measured metabolite concentrations can be found in Supplementary Tables 3, 4, and 5

### Effect of the KD on the gastrocnemius metabolome

**Fig. 2:**
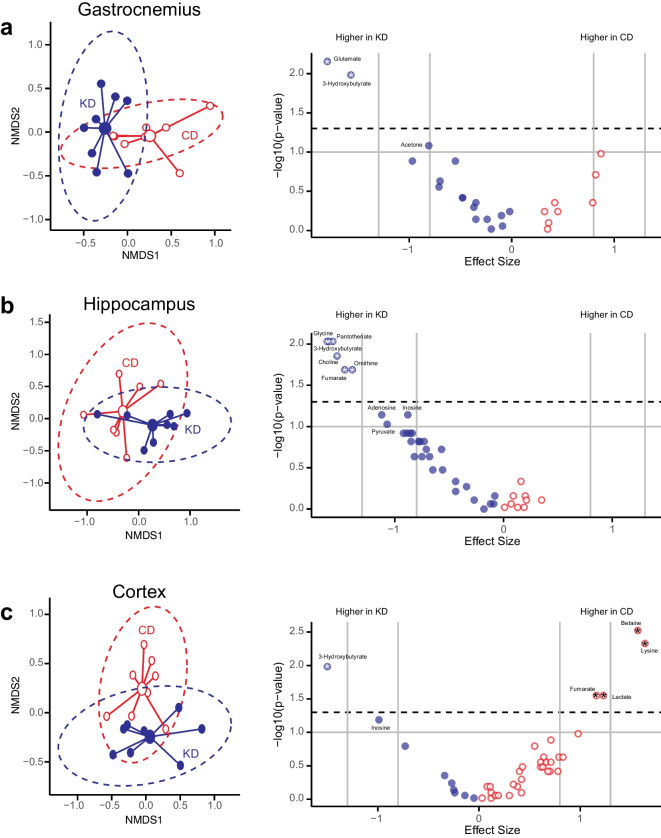
Impact of a 2-month ketogenic diet on the gastrocnemius, hippocampus, and cortex metabolomes. Non-metric multidimensional scaling (NMDS) plots (left) of log transformed data with 95% confidence ellipses and corresponding metabolite Volcano plots of Hedge’s *g* effect sizes versus log-transformed *p*-values (right) in **a** the gastrocnemius (*p* = 0.0063, stress = 0.028), **b** the hippocampus (*p* = 0.0379, stress = 0.048), and **c** the cortex (*p* = 0.01, stress = 0.037). Mice in the control group (CD) are indicated in red, and mice in the ketogenic diet group are indicated in blue. In the volcano plots, metabolites higher than the horizontal dotted line have *p*-values < 0.05, and those above the horizontal grey line have *p*-values < 0.1. The vertical grey lines represent Hedge’s *g* (*g*) cutoffs for large (|*g*|= 0.8) and very large (|*g*|= 1.2) effect sizes. A negative effect size indicates that the metabolites are larger in mice on the ketogenic diet (KD, blue) whereas a positive effect size indicates metabolites are larger in mice on the control diet (CD, red). Metabolites with an asterisk are considered significant (large or very large effect size and *p*-value < 0.05). Metabolites trending toward significance (large or very large effect size and *p*-value < 0.1) are also indicated

There was a statistically significant difference by group in the quantified gastrocnemius metabolites (PERMANOVA; *R*^2^ = 0.21, *p* = 0.0063 under 9999 permutations, Fig. 2a, Table 3). βHB, an energy source for skeletal muscle during ketosis, was significantly higher in the gastrocnemius of KD mice. We observed significantly higher glutamate and a trend for higher glycine was also noted. Alanine tended to be lower in the gastrocnemius of KD mice. Gastrocnemius glycine and formate were not correlated overall (*p* = 0.4632, rho =  − 0.1971, Supplementary Fig. 2), with no correlation in the CD group (*p* = 0.3268, rho =  − 0.4048) or the the KD group (*p* = 0.6169, rho =  − 0.2143).

### Effect of the KD on the hippocampal metabolome

PERMANOVA on the quantified metabolites indicated that there was a significant difference between groups in the hippocampal metabolome (*R*^2^ = 0.158, *p* = 0.0379 under 9999 permutations, Fig. 2b, Table 4). Higher βHB in the hippocampus of KD mice is consistent with expectations for mice on a KD diet. Other alterations induced by the KD included higher glycine, elevated acetyl-CoA precursors pantothenate and choline, higher tricarboxylic acid (TCA) and urea cycle intermediate fumarate, and elevated urea cycle metabolite ornithine.

**Table 4:** Metabolites significantly different or trending toward a difference between mice on either the control (CD) or ketogenic (KD) diet in the hippocampus and cortex (expressed as nmol/g). For each metabolite, median and interquartile range (IQR), Wilcoxon-rank sum *p*-value (*p*), and Hedge’s g effect size (*g*) are indicated

Metabolites	Hippocampus				Cortex				
	CD, median (IQR)	KD, median (IQR)	*p*	*g*	CD, median (IQR)		KD, median (IQR)	*p*	*g*
β-Hydroxybutyrate	69.7 (54.9–91.2)	200.1 (131.2–303.7)	0.0093*	− 1.57	91.6 (48.2–114)		203.8 (156.2–311.8)	0.0104*	− 1.5
Adenosine	136.6 (125.6–140.9)	205.4 (156.2–236.7)	0.0721^†^	− 1.12	-		-	-	-
Betaine	-	-	-	-	155.6 (140.3–172.5)		123.5 (116.3–129.5)	0.0030*	1.57
Choline	88.2 (75.1–95.2)	113.4 (111.5–117.6)	0.0140*	− 1.53	-		-	-	-
Fumarate	45.6 (44.1–54)	66.8 (62.5–72.9)	0.0205*	− 1.46	54.6 (52–57.9)		46.7 (44.5–49.1)	0.0281*	1.16
Glycine	819.3 (766.5–966.3)	1055.5 (985.5–1113.6)	0.0093*	− 1.61	-		-	-	-
Inosine	56.4 (53.5–62.3)	68.6 (63.1–74.3)	0.0721^†^	− 0.88	52.3 (50.1–73)		75.2 (63–80.1)	0.065^†^	− 0.99
Lactate	-	-	-	-	11,351.6 (9852.1–12,578)		9429.1 (8223.6–10,107.6)	0.0281*	1.23
Lysine	-	-	-	-	153.4 (147.6–170.8)		126.1 (119.3–130.4)	0.0047*	1.64
Ornithine	59.2 (53.6–66)	78.3 (72.5–89.4)	0.0205*	− 1.39	-		-	-	-
Pantothenate	32.5 (28.6–45.4)	51.1 (47.2–56.8)	0.0093*	− 1.62	-		-	-	-

*Metabolites significantly different between mice on the ketogenic diet vs. mice on the control diet (*p* < 0.05 and |*g*|≥ 0.8)

^†^Metabolites that trended toward a difference between mice on the ketogenic diet vs. mice on the control diet (*p* < 0.1 and |*g*|≥ 0.8)

-Metabolites not measured

All measured metabolite concentrations can be found in Supplementary tables 6 and 7

### Effect of the KD on the cortex metabolome

Based on the quantified metabolites in the cortex, we observed a statistically significant difference by group (PERMANOVA; *R*^2^ = 0.16, *p* = 0.01 under 9999 permutations, Fig. 2c, Table 4). Consistent with the hippocampus, we observed significantly higher βHB. We also observed significantly lower lactate, an energy metabolite produced in astrocytes from glucose. Significantly lower concentrations of the essential amino acid lysine, as well as fumarate and betaine, were also observed.

## Discussion

In this study, we analyzed the impact of a 2-month KD on metabolism in middle-aged (16-month-old) female mice. Much research has focused on the effects of a KD as a therapeutic for epilepsy [18], neurodegenerative disorders [19, 20], weight loss [21], metabolic disease [22], and cancers [23, 24]. There are limited studies exploring the impacts of a KD on the metabolome in rodents. Of those conducted, metabolomics research has focused on the liver [25], serum, and hippocampus [26] in male rodents. This study adds to the literature by showing the system-wide impact of a KD on middle-aged female mice, with many tissue-specific alterations in energy metabolism detected. We also discuss several potential mechanisms through which the KD may contribute to age-related benefits that provide opportunities for future exploration.

### A ketogenic diet impacts energy and fat metabolism

Previously, we reported that serum ketone bodies tended to decrease with age in female mice [27], consistent with impaired hepatic fatty acid oxidation with advancing age [28]. In this study, a 2-month KD in middle-aged female mice resulted in elevated βHB in serum, the liver, the gastrocnemius muscle, and the hippocampal and cortex brain regions, as well as higher concentrations of hepatic glycerol, consistent with the ketogenic diet phenotype previously described [29]. We also observed lower fasting serum glucose in KD mice compared to controls (after a 12-h fast). This is consistent with a previous study that showed that C57BL/6 male mice on a KD for 4 months had reduced blood glucose concentrations after a 6-h fast [25].

Lower serum taurine concentrations have been observed in aged mice and humans, while taurine supplementation has been shown to support health span in mice and liver function in aged non-human primates [30]. We observed lower hepatic taurine concentrations in mice in the KD group. However, no difference in serum taurine was observed between groups (Supplementary Table 1). Taurine and glycine are used in the conjugation of bile salts, with 95% of bile acids conjugated to taurine in mice [31]. However, the hepatic bile acid taurocholate did not differ between groups, suggesting that lower taurine concentrations are unlikely to be due to enhanced bile acid synthesis in KD mice. Since mitochondria-based energy metabolism is increased on a KD, it could be that hepatic taurine utilization is enhanced to support mitochondrial function and suppress oxidant generation [32]. Given that taurine concentrations did not differ between groups in serum or the other tissues assessed, it could be that renal reuptake of taurine is enhanced.

Carnitine is responsible for the transport of acyl groups across the mitochondrial membrane for β-oxidation of fatty acids [33]. Lower carnitine was noted in the kidney of KD mice compared to controls, while carnitine synthetic precursors, lysine and methionine, were not different (Supplementary Table 3). Carnitine concentrations are maintained in the body primarily through liver-based synthesis, provision from the diet, and renal reabsorption [34]. One explanation for this observation may be related to the fact that the primary energy source for the kidney is fatty acids [35]. Lower free carnitine could mean that a larger proportion of carnitine was converted to acyl-carnitine for transport across the mitochondrial membrane for β-oxidation. However, the physiological significance of low renal carnitine in KD mice remains to be determined.

In our study, we observed higher concentrations of leucine, isoleucine, and valine in the liver, but not in the serum or gastrocnemius muscle of mice on a KD (Supplementary Tables 1 and 4). Elevated liver branched-chain amino acids (BCAAs) is consistent with findings in 1-month old C57BL/6 J male mice on a KD for 4 month [25], suggesting these differences were unlikely to be related to sex or age. This hepatic accumulation of BCAAs suggests a reduced need to convert BCAAs to ketones or glucose. This makes sense as a KD primarily supports energy needs through fatty acid oxidation, while gluconeogenesis can be supported by increased glycerol availability. Additionally, BCAAs have been observed to induce fatty acid oxidation [36], which occurs primarily in the liver.

### A ketogenic diet impacts 1-carbon metabolism in liver and kidney

**Fig. 3:**
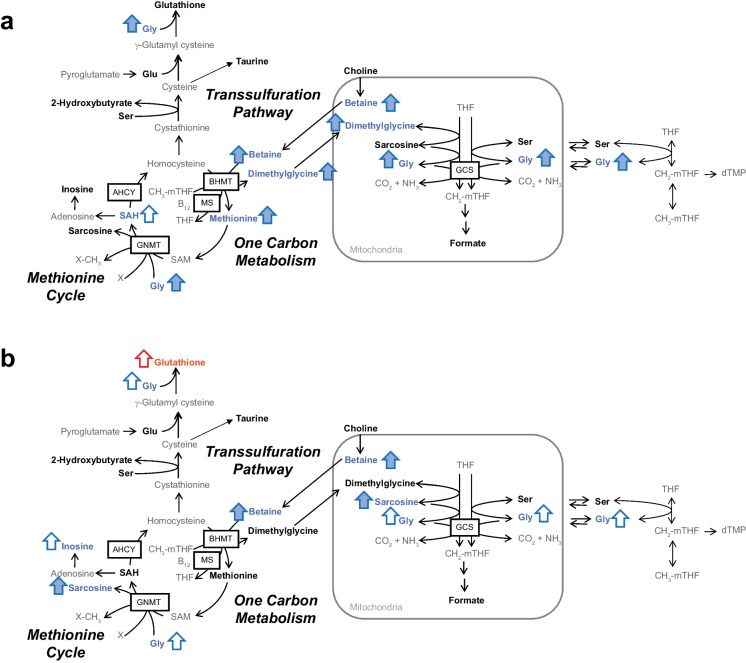
Effect of 2-month ketogenic diet on one-carbon metabolism, methionine cycle, and transsulfuration pathway in **a** the liver and **b** the kidney. Metabolites in bold were quantified using ^1^H-NMR. Blue metabolites and arrows indicate the metabolites are higher in mice on a ketogenic diet, whereas those in red are higher in mice on the control diet. Metabolites were compared using a Wilcoxon-rank sum test. Effect size was calculated using Hedge’s *g* (*g*). Filled arrows indicate statistical significance (*p* < 0.05 and |*g*|≥ 0.8) and unfilled arrows indicate a statistical trend (*p* < 0.1 and |*g*|≥ 0.8). Abbreviations: AHCY, adenosylhomocysteinase; B_12_, vitamin B12; BHMT, betaine homocysteine S-methyltransferase; CH_2_-mTHF, 5,10-methylene-THF; CH_3_-mTHF, 5-methyl-THF; dTMP, deoxythymidine monophosphate; GNMT, glycine N-methyltransferase; GCS, glycine cleavage system; Gly, glycine; Glu, glutamate; MS, methionine synthase; SAH, S-adenosylhomocysteine; SAM, S-adenosylmethionine; Ser, serine; THF, tetrahydrofolate; X, unmethylated species; X-CH_3_, methylated species

One-carbon metabolism is essential for cellular physiology, as it is involved in methylation, nucleotide biosynthesis, amino acid homeostasis, and the maintenance of redox balance. It encompasses three sub-pathways: the methionine cycle, the folate cycle, and the transsulfuration pathway. In our middle-aged female KD mice, we observed elevated concentrations of methionine cycle intermediates betaine, dimethylglycine (DMG), and methionine, as well as a significantly higher betaine:DMG ratio (Supplementary Fig. 3) and a trend for higher S-adenosylhomocysteine (SAH) in the liver. A growing body of evidence suggests that βHB can regulate gene expression through β-hydroxybutyrylation (*Kbhb*) of histone protein lysine residues and can regulate enzyme activity through *Kbhb* of non-histone proteins in order to regulate specific metabolic pathways [37]. Interestingly, one of the pathways enriched by *Kbhb* of lysine residues is one-carbon metabolism [37]. There are two reactions in the methionine synthetic pathway (Fig. 3a) that can convert homocysteine (HCy) to methionine. The first involves betaine-homocysteine S-methyltransferase (BHMT), which converts Hcy to methionine. Activity of this enzyme appears to be attenuated in the liver, as we observed a higher betaine:DMG ratio. However, methionine is higher in concentration, which suggests that the alternative enzyme (methionine synthase (MS)) that converts Hcy to methionine is activated. We also observed a trend toward higher SAH, suggesting attenuation of the enzyme adenosylhomocysteinase (AHCY), the rate-limiting enzyme of the methionine cycle, which converts SAH to Hcy. This is consistent with previous findings that have shown that *Kbhb* inhibits AHCY activity [37]. Protein acetylation is another mechanism of regulation. It was recently reported that the KD increases global lysine acetylation in the liver, but more work is needed to determine which proteins show increased acetylation with a KD and the influence of acetylation on the function of these proteins [38].

We observed higher glycine in the liver and serum (trend) in the KD group compared to controls. While there was no difference between groups in liver or serum (Supplementary Tables 1 and 2) formate, a strong positive association between liver glycine and formate was observed in CD, but not KD mice (Supplementary Fig. 1). These results suggest that hepatic glycine is tied to formate production in CD, but not in KD mice, and is consistent with a previous study where it was observed that a KD reduced activity of the mitochondrial glycine cleavage system (GCS) thus limiting formate release from mitochondria [39]. Elevated glycine could also be related to modulation of carbamoyl phosphate synthetase 1 (CPS1), the rate-limiting enzyme in the urea cycle that converts GCS generated NH_4_^+^ to carbamoyl phosphate, which has shown to be highly enriched by *Kbhb* in the liver of fasted mice [37].

In agreement with our results in the liver, we observed significantly higher betaine, and a trend for higher glycine in the kidney of KD mice, but no differences in DMG or methionine were observed (Fig. 3b, Table 3, Supplementary Table 3). We also observed sarcosine was elevated and glutathione trended lower in the kidney. If *Kbhb* plays an important role in one carbon metabolism, these results suggest that the pattern of *Kbhb* may be different in different organs. Indeed, it has been shown that *Kbhb* is widespread in the kidney similar to the liver, but not pancreatic, skeletal muscle, colon, heart, or cerebral cortex of fasted compared to fed mice [37].

### A ketogenic diet impacts renal osmolytes

Organic osmolytes play an important role in regulating osmotic pressure and maintaining protein structure and function in the inner medulla of the kidney [40, 41]. In a prior study on rats, sorbitol, glycerophosphocholine (GPC), and myo-inositol were elevated in the renal medulla in order to compensate for osmotic changes as a result of dehydration [42]. During the first few weeks after initiating a ketogenic diet, water loss has been reported, which has been attributed to depletion of water-associated glycogen stores [43]. However, less is known about the long-term effects of a ketogenic diet on water and electrolyte handling in the kidney. Interestingly, we observed higher betaine and myo-inositol (trend), but not GPC or sorbitol (Supplementary Table 3), in whole kidney of the KD compared to the CD group. Thus, additional work is needed to determine the impact of these changes in betaine and myo-inositol on electrolyte balance and kidney function.

### A ketogenic diet impacts the glucose-alanine cycle

**Fig. 4:**
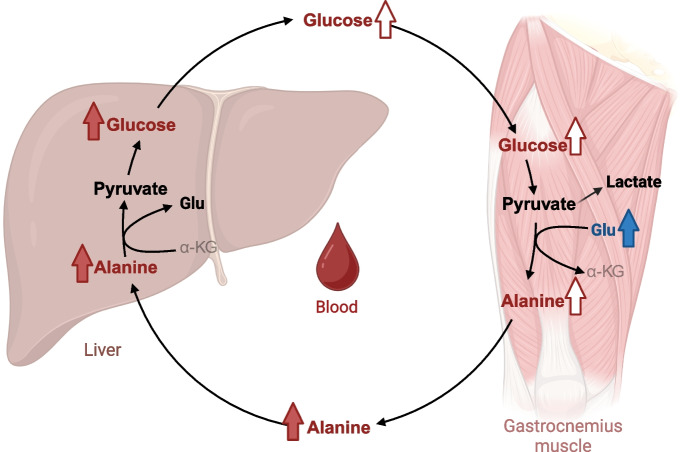
Effect of 2-month ketogenic diet on the glucose-alanine cycle in the liver, serum, and gastrocnemius muscle. Metabolites in bold were quantified using ^1^H-NMR. Metabolites and arrows in red are higher in mice in the control group, whereas those in blue are higher in mice in the ketogenic diet group. Metabolites were compared using a Wilcoxon-rank sum test in the liver and serum. Log_10_-transformed metabolites were compared using a Welch’s *t*-test in gastrocnemius muscle with the exception of lactate, which was compared using a Wilcoxon rank-sum test, as it could not be normalized by log_10_-transformation. Effect size was calculated using Hedge’s g (*g*). Filled arrows indicate statistical significance (*p* < 0.05 and |*g*|≥ 0.8) and unfilled arrows indicate a statistical trend (*p* < 0.1 and |*g*|≥ 0.8). Abbreviations: α-KG, α-ketoglutarate; Glu, glutamate. This figure was generated using BioRender

In gastrocnemius muscle, middle-aged KD females experienced higher glutamate and a trend for lower alanine, an important hepatic gluconeogenic precursor during prolonged fasting-induced muscle protein degradation. These findings suggests that the KD suppresses glutamate transamination via inhibition of alanine aminotransferase (Fig. 4). Ketone bodies have been shown to inhibit alanine production from BCAAs and alanine release from skeletal muscle in rats [44, 45] and chicks [46], which is consistent with lower alanine in gastrocnemius muscle (trend), serum, and the liver observed in this study. In alignment with reduced alanine in circulation, glucose concentrations were also lower in the liver and serum (trend) of KD mice. Since both KD and CD mice were equivalently fasted prior to necropsy, these results support the notion that a 2-month KD alters the glucose-alanine cycle in middle-aged females.

### A ketogenic diet impacts brain metabolism

The brain relies predominantly on glucose for energy metabolism. However, other metabolites such as lactate, ketone bodies, and acetate are also used to support energy needs [47]. In our mice, the impact of a KD on brain metabolism differed depending on the brain region. For instance, while we observed twofold higher βHB in the hippocampus and cortex of KD mice compared to CD mice, we observed lower lactate only in the cortex of KD mice. Since we did not detect a decrease in pyruvate in the cortex (Supplementary Table 7), these results could suggest there may be reduced lactate dehydrogenase activity in astrocytes, as lactate can be produced downstream from glucose to support energy needs in neurons. Thus, given that we observed no difference in adenosine triphosphate (ATP) in either brain region, these results suggest that energy production was not affected by this shift in metabolism.

**Fig. 5:**
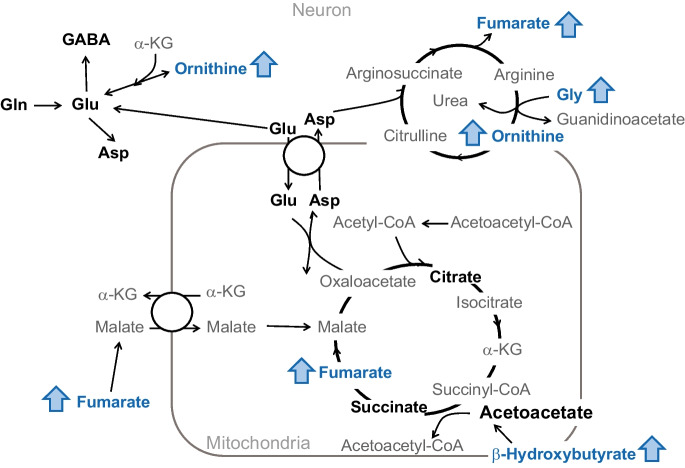
Effect of a 2-month ketogenic diet on the tricarboxylic acid (TCA) cycle and urea cycle in the hippocampus. Metabolites in bold were quantified using ^1^H-NMR. Metabolites and arrows in red are higher in mice in the control group, whereas those in blue are higher in mice in the ketogenic diet group. Metabolites were compared using a Wilcoxon-rank sum test. Effect size was calculated using Hedge’s g (*g*). Filled arrows indicate statistical significance (*p* < 0.05 and |*g*|≥ 0.8) and unfilled arrows indicate a statistical trend (*p* < 0.1 and |*g*|≥ 0.8). Abbreviations: α-KG, α-ketoglutarate; Asp, aspartate; GABA, γ-aminobutyric acid; Gln, glutamine; Glu, glutamate

Paradoxically, we observed higher fumarate in the hippocampus and lower fumarate in the cortex of KD mice compared to CD mice. This metabolic divergence could be related to differences in tricarboxylic acid (TCA) cycle activity. However, we did not detect any differences in other TCA metabolites (Supplementary Table 6). Fumarate is also a known agonist of nuclear factor erythroid 2 p45-related factor 2 (*Nrf2*) [48] which has been shown to improve synaptic plasticity, learning, and memory in the hippocampus [49, 50]. These results suggest that elevations in hippocampal fumarate may contribute to increased antioxidant defenses, which has been previously reported after a 3-week KD [51]. Additionally, higher glycine and ornithine were also detected in KD mice. Since fumarate, glycine, and ornithine are all involved in the urea cycle (Fig. 5), these results indicate that a KD might alter the urea cycle in the hippocampus. This alteration could be associated with increased protein turnover within this brain region, as myelin sheath proteins have been shown to have a fast turnover in the brain [52]. A growing body of evidence has shown that myelinogenesis is essential for a supporting memory function and that oligodendrocyte myelinogenesis declines with advancing age [53]. Thus, future investigation into changes to the urea cycle due to a KD might support enhanced myelin turnover and long-term memory.

An interesting difference between the hippocampus and the cortex was that we observed higher levels of hippocampal myelin cofactors pantothenate and choline. Choline is an essential nutrient. Pantothenate is a vitamin necessary for production of acetyl-CoA, which is a key substrate for fatty acid and cholesterol synthesis, which are essential for myelin formation [54]. Recently, it was shown that large pantothenate stores are localized in myelinated structures of the brain, indicating a significant need for accessible pantothenate to support myelin synthesis to preserve myelinated neuron integrity [55]. The hippocampus plays an essential role in the formation of episodic memories, the memories that allow the storing of information about time, place, and other details about events [56]. Neural activity has been shown to regulate oligodendrogenesis, the creation of cells responsible for myelination of axons, the neural projections that facilitate communication between brain regions [57]. In particular, spatial learning in both humans and rats has been reported to alter the microstructure of white matter tracts in the fornix, the major output tract of the hippocampus [58]. In the middle-aged female mice, it was previously reported that a KD improved spatial memory [12]. Thus, higher hippocampal levels of pantothenate and choline could suggest enhanced de novo myelination of surrounding white matter tracts, which might aid in spatial memory preservation [58].

## Conclusions

Our results on the ketogenic metabolic phenotype of female mice were similar to previous findings in male mice [7, 17], including elevated ketone body production and markers of increased fatty acid catabolism. Alterations in skeletal muscle and liver metabolism suggests that a KD modulated the glucose-alanine cycle. Intermediates of one-carbon metabolism and the methionine cycle were differentially altered in the liver and kidney, which may be regulated by post-translational modifications by *Kbhb*. Novel findings include elevations in renal osmolytes betaine and myo-inositol (trend) in the KD group, and the distinct metabolic differences observed across hippocampal and cortex tissues. Indeed, several changes noted in the hippocampus could relate to the beneficial effects of a KD on age-related changes in memory in middle-aged female mice.

## Supplementary Information

Below is the link to the electronic supplementary material.

Supplementary file1 (PDF 419 KB)

## Data Availability

Data are available upon reasonable request.

## Abbreviations

*ATP*: Adenosine triphosphate
*AHCY*: Adenosylhomocysteinase
*α-KG*: α-Ketoglutarate
*AIN-93G*: American Institute of Nutrition, 1993, growth, pregnancy and lactation
*Asp*: Aspartate
*BHMT*: Betaine homocysteine S-methyltransferase
*BCAA*: BRANCHED-chain amino acids
*βHB*: β-Hydroxybutyrate
*Kbhb*: β-Hydroxybutyrylation
*CPS1*: Carbamoyl phosphate synthetase 1
*CD*: Control diet
*dTMP*: Deoxythymidine monophosphate
*GABA*: G-Aminobutyric acid
*Glu*: Glutamate
*Gln*: Glutamine
*GPC*: Glycerophosphocholine
*Gly*: Glycine
*GCS*: Glycine cleavage system
*GNMT*: Glycine N-methyltransferase
*g*: Hedge’s *g*
*Hcy*: Homocysteine
*KD*: Ketogenic diet
*MS*: Methionine synthase
*CH*_*2*_-*mTHF*: 5,10-Methylene-THF
*X*-*CH*_*3*_: Methylated species
*CH*_*3*_-*mTHF*: 5-Methyl-THF
*DMG*: N,N-Dimethylglycine
*NIA*: National Institute of Aging
*NIH*: National Institute of Health
*NMDS*: Non-metric multi-dimensional scaling
*Nrf2*: Nuclear factor erythroid 2 p45-related factor 2
*NMR *: Nuclear magnetic resonance
*PERMANOVA*: Permutational multivariate analysis of variance
*SAH*: S-Adenosylhomocysteine
*SAM*: S-Adenosylmethionine
*Ser*: Serine
*TBHQ*: Tert-Butylhydroquinone
*THF*: Tetrahydrofolate
*TCA*: Tricarboxylic acid
*UC*: University of California
*X*: Unmethylated species
*B*_*12*_: Vitamin B12
