# Supplementary material for: Two-month ketogenic diet alters systemic and brain metabolism in middle-aged female mice
Source: GeroScience. 2024 Aug 24;47(1):935–52. doi: 10.1007/s11357-024-01314-w (PMC11872878; doi:10.1007/s11357-024-01314-w)
Supplement: Supplementary file 1 — Supplementary file1 (PDF 419 KB) [file 11357_2024_1314_MOESM1_ESM.pdf]

SUPPLEMENTARY INFORMATION

Two-month ketogenic diet alters systemic and brain metabolism in middle-aged female mice

GeroScience

Kirsten J. Roslund, Jon J. Ramsey, Jennifer M. Rutkowsky, Zeyu Zhou and Carolyn M. Slupsky

Corresponding Author:

Carolyn M. Slupsky

Department of Nutrition and Department of Food Science & Technology

University of California, Davis

Davis, CA. 95616

e-mail: cslupsky@ucdavis.edu

Liver

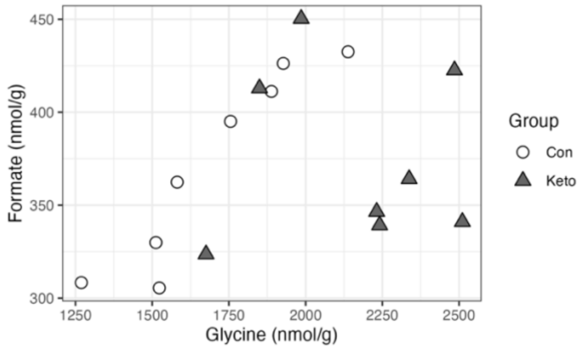

Supplementary Fig. 1 Spearman correlation between liver glycine and formate in mice on the control diet (open circles, Con), and mice on the ketogenic diet (closed triangles, keto). Overall correlation, *p* = 0.0822, rho = 0.4500. Correlation of mice on the ketogenic diet, *p* = 0.8401, rho = 0.0952. Correlation of mice on the control diet, *p* = 0.0022, rho = 0.9286

Gastrocnemius

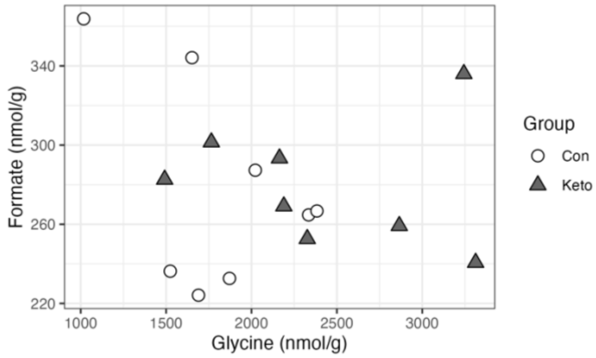

Supplementary Fig. 2 Spearman correlation between gastrocnemius glycine and formate in mice on the control diet (open circles, Con), and mice on the ketogenic diet (closed triangles, keto). Overall correlation, *p* = 0.4632, rho = -0.1971. Correlation of mice on the ketogenic diet, *p* = 0.3268, rho = -0.4048. Correlation of mice on the control diet, *p* = 0.6169, rho = -0.2143

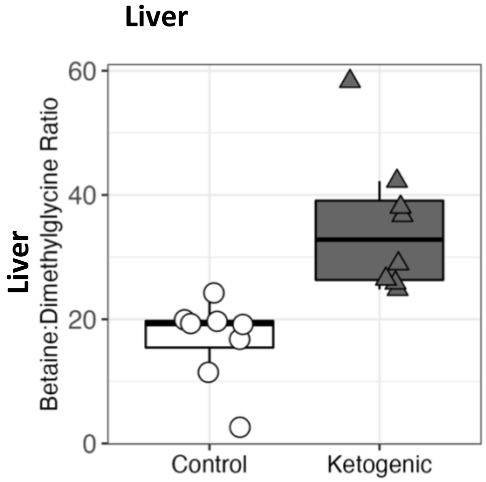

Supplementary Fig. 3 Comparison of the ratio of liver betaine to dimethylglycine using the Wilcoxon-rank sum test and Hedge's g (*g*) effect size in mice on the control diet (open circles), and mice on the ketogenic diet (closed triangles). $p = 0.0002$, $g = -1.88$

Supplementary Table 1 Median serum metabolite concentrations (µM), and comparison between mice on a KD vs CD using the Wilcoxon-rank sum test and Hedge's g effect size measure. For each metabolite, median and interquartile range (IQR), p-value (*p*), and Hedge's g effect size (*g*) are indicated. Metabolites considered significantly different have a $p < 0.05$, and at least a large effect size, $|g| \geq 0.8$

**Metabolite**	**Control, Median (IQR)**	**Ketogenic, Median (IQR)**	***p***	***g***
2-Aminobutyrate	27 (25.5-33.2)	24.6 (20.4-27.1)	0.3282	0.65
2-Hydroxybutyrate	45.4 (41.8-59.2)	55.7 (49.5-62.6)	0.4418	-0.52
2-Hydroxyisobutyrate	5.1 (3.7-7.1)	3.7 (3.2-4.9)	0.2345	0.59
2-Hydroxyisovalerate*	6 (5.3-6.5)	7.7 (6.4-8.5)	0.0281	-1.21
2-Oxoglutarate	28.4 (23.1-33.4)	20.3 (16-24.8)	0.1605	0.57
2-Oxoisocaproate*	5.1 (3.3-6.7)	3 (2.1-3.7)	0.0499	1.17
β-Hydroxybutyrate*	867 (527.4-1391)	2006.3 (1372.1-2538.3)	0.0148	-1.46
3-Hydroxyisobutyrate	22.9 (15.3-26.4)	22.8 (20.1-30.3)	0.5737	-0.36
3-Hydroxyisovalerate	1.3 (1-1.7)	1 (0.8-1.2)	0.1605	0.77
3-Methyl-2-oxovalerate	7 (6.3-9)	4.8 (4-5.6)	0.1605	0.68
Acetate	104.1 (92.5-111.1)	95.7 (87.3-104.6)	0.5737	0.22
Acetone	1.8 (1-3.2)	2.5 (0.4-4.3)	0.9591	-0.34
Alanine*	466.5 (391.1-476)	286 (225-358.9)	0.0104	1.61
Arginine	121.5 (103.9-128.8)	92.3 (81.4-110.4)	0.4418	0.28
Asparagine	65 (53.4-72.9)	67 (52.3-72.8)	0.9591	-0.01
Aspartate	18.8 (10.8-20.6)	12.5 (9.2-14.1)	0.2345	0.41
Betaine*	58 (53.9-67.7)	87 (82.6-99.9)	0.0070	-1.09
Butyrate	5.9 (5.1-7.4)	6.2 (5-6.6)	0.7209	0.31
Choline	20.3 (16.5-22.2)	17.6 (14.5-20.3)	0.6454	0.43
Citrate	314 (299.6-338.9)	275.6 (256.6-316.9)	0.2345	0.38
Creatine*	149 (141-168.9)	118 (104.6-133.7)	0.0379	1.19
Creatinine	7.7 (5.6-9.4)	6.3 (5.6-6.9)	0.6454	-0.04
Dimethylamine	1.2 (1-1.4)	1.4 (1.1-1.6)	0.4418	-0.41
Formate	11 (10.6-13)	13.9 (9.6-16.5)	0.5054	-0.50
Fumarate	2.9 (2.1-4.8)	1.9 (1.7-3.3)	0.1949	0.57
Glucose†	10144.6 (9892.5-10899.2)	7808.6 (7374.1-9710.1)	0.0830	0.96
Glutamate	76.6 (68.3-80.6)	59.2 (50.2-73.8)	0.1304	0.48
Glutamine	601.9 (589.5-661.1)	630.6 (572.7-668.9)	>0.9999	-0.03
Glycerol	1036.3 (831.8-1092.4)	822.8 (740.1-878.4)	0.3282	0.27
Glycine†	223.3 (204.5-237)	273 (227.1-315.1)	0.0650	-1.06

Histidine	42.3 (36.5-45.1)	33.3 (31.6-37.3)	0.1605	0.71
Isoleucine	96.9 (86.6-108.7)	96.1 (82.7-100.2)	0.7984	0.17
Lactate	3522.2 (3237.4-3833.4)	3356.7 (2790-3589.1)	0.3823	0.48
Leucine	122.6 (101.8-134.7)	119.6 (101.5-126.6)	0.7984	-0.36
Lysine*	211.7 (201.4-236.1)	188.4 (166.7-206.1)	0.0379	1.06
Methanol	4.4 (3.4-5.2)	3 (2.5-3.9)	0.2786	0.57
Methionine	54.6 (43.7-57.9)	48.9 (47.7-50.1)	0.4418	0.45
Myo-inositol	26.1 (17.9-32)	31.8 (19.1-47.3)	0.3823	-0.63
Dimethylglycine	4.9 (4.6-5.4)	9.9 (8.5-10.7)	0.0054	-1.72
O-Acetylcarnitine	30.3 (27.8-32.4)	29 (27.8-29.7)	0.5054	0.07
Ornithine	79.9 (63.9-85.6)	81.6 (56.3-86.1)	0.6454	-0.33
Phenylalanine	52.4 (46.5-57.1)	49.2 (47.2-54.7)	0.8785	-0.03
Proline	95.9 (78.3-124.7)	98.9 (81.2-117.5)	0.8785	0.06
Pyruvate*	109.8 (94.3-120.1)	68.2 (60.5-79.9)	0.0104	1.55
Serine	153.4 (132.7-178.4)	141 (112.6-171.3)	0.7209	0.07
Taurine*	808.8 (683.3-874.6)	566.9 (463.4-655.9)	0.0207	1.38
Threonine	166.3 (160.2-172.1)	161.5 (129-168.3)	0.3282	0.71
Trimethylamine N-oxide	6.5 (4.9-8.1)	5.3 (4.2-6.6)	0.5737	0.05
Tyrosine	75.9 (68.3-81.3)	82.7 (74.6-90.7)	0.3823	-0.40
Valine	191.5 (162.2-200.4)	161.2 (144.1-173.1)	0.2345	0.38

*Metabolites that were significantly different after comparison
†Metabolites that trended toward a difference after comparison

Supplementary Table 2 Median liver metabolite concentrations (nmol/g), and comparison between mice on a KD vs CD using the Wilcoxon-rank sum test and Hedge's g effect size measure. For each metabolite, median and interquartile range (IQR), p-value (*p*), and Hedge's g effect size (*g*) are indicated. Metabolites considered significantly different have a $p < 0.05$, and at least a large effect size, $|g| \geq 0.8$

**Metabolite**	**Control, Median (IQR)**	**Ketogenic, Median (IQR)**	***p***	***g***
2-Aminobutyrate	76.9 (66.7-89)	88.7 (75.7-92.4)	0.3823	-0.40
2-Hydroxybutyrate	48.4 (38-51.6)	55.3 (49.9-58.5)	0.1559	-0.68
2-Oxoisocaproate	6.4 (5.6-8.7)	7.7 (7.3-9.1)	0.2268	-0.62
β-Hydroxybutyrate*	508.9 (331.6-837.1)	1129.8 (807.6-1373.5)	0.0148	-1.50
3-Hydroxyisobutyrate	13.2 (12.4-18.5)	18.7 (16.3-21.1)	0.1049	-0.87
3-Hydroxyisovalerate	6.3 (5.5-6.9)	6.3 (4.1-7.2)	0.9581	0.11
ADP	44.2 (31.3-54.7)	55.7 (47.1-61.2)	0.3282	-0.37
AMP†	591.4 (530.7-756.3)	739.2 (719.3-842.2)	0.0830	-0.82
ATP	81.4 (66.4-86.4)	98.9 (75.2-111.7)	0.1033	-1.06
Acetate	219.5 (196.6-269.2)	253.9 (216.3-270.2)	0.4418	-0.20
Acetoacetate	15.5 (10.8-20.4)	17.7 (14.1-20.4)	>0.9999	-0.01
Acetone	211.5 (184.5-222.4)	218.7 (212.3-223.4)	0.2786	-0.69
Alanine*	2680.2 (2281-2902.5)	2150.4 (2008.4-2434.3)	0.0379	1.10
Arginine	168.6 (79.2-207.5)	230.3 (169.9-283.2)	0.1304	-0.68
Ascorbate	189.7 (146.3-260.8)	172.7 (117.4-316)	0.9591	-0.12
Asparagine	114 (105.9-154.8)	140.4 (126.8-161.4)	0.5054	-0.51
Aspartate	420 (339-548.7)	466.6 (420.2-605.2)	0.5737	-0.12
Betaine*	326.9 (261.8-389.4)	948.3 (856.6-1250.9)	0.0002	-1.62
Butyrate	13.4 (9.1-17.7)	12.9 (12.6-15.9)	0.7927	-0.24
Carnitine	170.9 (149.2-297.1)	287.5 (184.4-372.7)	0.5054	-0.24
Choline	183.8 (168.6-226.6)	302.5 (193.9-312.9)	0.0830	-0.61
Creatine	266.2 (237.1-305.5)	362 (266.9-387.3)	0.1949	-0.73
Creatinine	19.1 (17.6-28)	21.5 (19.8-25.7)	0.7984	0.16
Formate	378.7 (324.5-415)	355.3 (340.6-415.3)	0.7984	-0.07
Fumarate	64.2 (56.2-71.4)	80.3 (75.6-87.4)	0.1049	-0.52
Glucose*	39599.2 (37123.6-41866.3)	32085.5 (27953.6-33430.4)	0.0006	1.65
Glutamate	968.4 (804.3-1167.4)	1115.9 (1038-1999.2)	0.1304	-0.67
Glutamine	4513.5 (3969.8-5239.9)	5108.5 (4518.6-5506.7)	0.2345	-0.58
Glutathione	935.5 (656.3-1012.9)	745.9 (660.8-815.1)	0.5054	0.34
Glycerol*	185.5 (162.9-201.6)	308.4 (272.4-364.7)	0.0003	-2.03

Glycine*	1668.4 (1520.4-1898.2)	2236.3 (1951.5-2374.2)	0.0104	-1.51
Histidine	324.7 (321.2-356.6)	364 (323-378.6)	0.7209	-0.08
Inosine	168.3 (159.8-194.5)	162.8 (145.5-174.7)	0.3823	0.21
Isoleucine*	140.3 (126.1-151.8)	172.9 (151.7-204.3)	0.0379	-1.04
Lactate	7479.7 (6503.8-8635.2)	7228.9 (6753.7-7912.8)	0.7209	0.26
Leucine*	242.1 (210.9-283.7)	324.4 (269-369.4)	0.0148	-1.12
Lysine	269.3 (211.7-295.1)	245 (220.1-290.7)	0.9591	0.19
Methionine*	29.9 (28.5-34.2)	46.1 (40.8-50.7)	0.0019	-1.92
Myo-inositol	290.9 (225-398.7)	405.5 (352.3-429.8)	0.1049	-0.92
Dimethylglycine*	18 (16.8-20.6)	32.7 (26.8-39.4)	0.0148	-1.10
NAD+	322 (289.6-394.8)	348.6 (314.6-365.6)	0.7984	-0.02
NADH	52.3 (47.4-54.6)	55.5 (46-58.8)	0.5737	-0.38
NADP+	49.3 (37.8-64.7)	73.8 (62-83.3)	0.1049	-0.39
Niacinamide	143.6 (121.4-162.5)	142.8 (112.9-151)	0.9591	0.02
O-Acetylcarnitine	76.8 (62.2-88.4)	71.1 (56.4-87.3)	0.7984	0.18
Ornithine	93.2 (70.5-125.6)	129.7 (101.7-156.4)	0.4418	-0.35
Phenylalanine†	92 (86.1-99.7)	103.4 (97.1-119.8)	0.0830	-0.92
Proline	117.3 (98.4-145.4)	175.8 (129.7-202.4)	0.1049	-0.84
Pyroglutamate	353 (324.7-485)	429.7 (388-462.5)	0.4418	-0.22
Pyruvate	21.5 (17.6-26.8)	19.5 (12.5-22)	0.2786	0.62
S-Adenosylhomocysteine†	26.4 (25.2-27.6)	28.5 (27.3-32.5)	0.0659	-0.80
Sarcosine	5.7 (5.1-7)	9.1 (8-9.8)	0.1949	-0.30
Serine	298 (275.5-403)	379.8 (375.5-437)	0.1605	-0.98
Succinate	841.2 (748.1-956.4)	985.5 (853.9-1038)	0.1605	-0.56
Taurine	22396.2 (20445-25627.3)	19735.3 (17298-21983)	0.1049	1.00
Taurocholate	140.7 (63.8-186.3)	184 (122.7-288.6)	0.4418	-0.49
Threonine	166.7 (126.3-198.3)	163.1 (138.4-198.2)	0.9591	0.30
Tyrosine*	129.7 (124.5-135.3)	161 (152.9-174.7)	0.0379	-1.27
Uridine	165.8 (157.2-172.4)	180.3 (141.3-193.7)	0.4418	-0.42
Valine*	224.2 (198.7-240.6)	260.1 (240.7-304.7)	0.0499	-0.97

*Metabolites that were significantly different after comparison
†Metabolites that trended toward a difference after comparison

Supplementary Table 3 Median kidney metabolite concentrations (nmol/g), and comparison between mice on a KD vs CD using the Wilcoxon-rank sum test and Hedge's g effect size measure. For each metabolite, median and interquartile range (IQR), p-value (*p*), and Hedge's g effect size (*g*) are indicated. Metabolites considered significantly different have a $p < 0.05$, and at least a large effect size, $|g| \geq 0.8$. Metabolites trending toward a difference have a $p < 0.1$, and at least a large effect size, $|g| \geq 0.8$

**Metabolite**	**Control, Median (IQR)**	**Ketogenic, Median (IQR)**	***p***	***g***
2-Aminobutyrate	54.7 (48.8-56.5)	51.1 (44.7-56)	0.7789	0.06
2-Hydroxybutyrate	31.4 (27.1-34.4)	32 (30.1-40)	0.6126	-0.32
2-Oxoisocaproate	5.4 (4.7-6)	5.1 (4.1-8.7)	>0.9999	-0.45
β-Hydroxybutyrate†	305.5 (169.1-742.3)	898.3 (571.5-1166.9)	0.0541	-1.11
3-Hydroxyisobutyrate	15.7 (13.7-16)	13.2 (12.8-20.1)	0.7789	-0.32
3-Hydroxyisovalerate	3.6 (3.1-5.3)	3 (2.8-4.3)	0.2810	0.38
AMP	1240.6 (1087.2-1410.8)	1321.3 (1182.9-1485.1)	0.3969	-0.34
Acetate	291.4 (234-428.8)	281.4 (261.2-328.2)	0.6943	0.22
Acetoacetate	9.1 (8.5-13.5)	14.1 (8.9-15.1)	0.3969	-0.43
Acetone	205.2 (181.1-214.7)	193.7 (191.7-196.1)	0.6126	0.40
Alanine	1172.2 (967.7-1278)	1001.7 (764-1077.1)	0.2319	0.77
Arginine	130.4 (110.6-205.1)	140.3 (125.2-150.1)	0.9551	0.24
Ascorbate	143.7 (122.3-158)	178.4 (138.9-191)	0.4634	-0.37
Asparagine†	108.7 (87.1-126)	131.9 (121.3-148.1)	0.0939	-0.90
Aspartate	1521 (1228.6-1610.1)	1511.9 (1223.2-1605.3)	0.6943	0.07
Betaine*	1060.1 (990.6-1077.2)	1428.4 (1359.8-1801.1)	0.0059	-1.73
Butyrate	14.5 (13-19.9)	13.8 (11.4-17.6)	0.8665	0.06
Carnitine*	209 (197.5-244.5)	144.5 (124.1-181.3)	0.0401	0.87
Choline	662.5 (510.1-688.1)	666.5 (597.8-716.3)	0.3357	-0.55
Citrate	238.6 (177-269)	185.5 (170.6-264.8)	0.8665	0.06
Creatine	1372.2 (1085.7-1650.6)	1260 (945.8-1637.6)	0.8665	0.16
Creatinine	33.8 (28.1-42.5)	36.6 (30.1-48.8)	0.8665	-0.21
Ethanolamine	398.6 (334.1-461.6)	457.5 (436.7-477.6)	0.1520	-0.74
Formate	432.3 (423.2-456.1)	402.3 (387.7-421.7)	0.1206	0.62
Fumarate	37.3 (30.2-48.3)	45.4 (40.9-53.2)	0.3969	-0.44
Sorbitol	697.1 (549.5-730.2)	857.5 (633.1-1026.1)	0.2319	-0.68
Glucose	3980.9 (3442.7-4715.6)	4104.3 (3688.7-4341)	>0.9999	0.03
Glutamate	3929.3 (3428.3-4197.1)	3937.4 (3468.2-4395.3)	0.6943	-0.28
Glutamine	1229.1 (1082.7-1260.1)	1330.3 (1234.4-1368.8)	0.2810	-0.35
Glutathione†	271.7 (231.8-315.4)	221.3 (129.5-243.1)	0.0939	0.98

Glycerol	354 (267.9-441.5)	383.5 (356.2-434.3)	0.6126	-0.48
Glycine†	2685.1 (2632.9-2881.9)	3395.1 (2894-3529.3)	0.0721	-1.22
Hypoxanthine	137.8 (103.8-177.4)	152.2 (142.6-189.5)	0.2810	-0.60
Inosine†	160.6 (130-189)	197.5 (180.9-240)	0.0939	-1.05
Isoleucine	110.9 (99.4-139.1)	136.2 (95.6-150.2)	0.6943	-0.34
Lactate	7216.2 (6274.4-8165.4)	6628.8 (5281.5-6931.1)	0.1893	0.41
Leucine	165.3 (144.7-194.3)	197.5 (137.7-210.1)	0.5358	-0.4
Lysine	165.6 (146.7-183.5)	179.6 (166.4-190.4)	0.3969	-0.38
Methionine	57.4 (53.6-69.4)	49.9 (46.2-65.2)	0.5358	0.22
Myo-inositol†	5673.3 (5259.4-5946.1)	7645.4 (5883.1-7992.5)	0.0721	-1.41
Dimethylglycine	2.5 (2-3.4)	2.9 (1.5-3.3)	0.7789	0.06
NAD+	597.3 (467.6-675.1)	667.6 (540-736.1)	0.4634	-0.45
NADH	37.3 (19-50.8)	36 (32.1-48.8)	0.6126	-0.17
NADP+	69.1 (65.6-95.7)	95.2 (67-114.4)	0.4634	-0.43
O-Acetylcarnitine	130.4 (107.3-150)	117 (109-127)	0.3969	0.54
O-Acetylcholine	41.6 (39.5-52.5)	57.1 (50.4-76.4)	0.2810	-0.44
O-Phosphocholine	608.4 (510.2-760.1)	614 (530.6-656)	0.6943	0.34
O-Phosphoethanolamine	2172.6 (1887.1-2336.5)	2169 (1922.4-2400.8)	0.9551	-0.19
Ornithine	53.4 (50-60.1)	52.8 (46.6-64)	0.8665	-0.06
Phenylalanine	58.4 (49.3-61)	63.4 (57.1-80.8)	0.1520	-0.91
Proline	123.7 (84.5-153.4)	122.9 (110.7-157.7)	0.5358	-0.36
Pyruvate	30.1 (23.8-37.9)	22.2 (19.9-28.4)	0.1206	0.83
Sarcosine*	6.2 (3.9-7.7)	9.5 (7.1-10.7)	0.0289	-1.29
Serine	442.2 (404.2-588.5)	404.8 (384.3-543)	0.6943	0.23
Glycerophosphocholine	7417.2 (6632.4-7871.3)	7682.8 (6559.5-8775.8)	0.4634	-0.40
Succinate	923.6 (790.4-1127.3)	986.8 (833.8-1049.6)	0.9551	-0.10
Taurine	14514.1 (12666-15027.6)	15614.9 (13312.3-17072.3)	0.2319	-0.62
Threonine	223.4 (185.6-233.8)	253.5 (232.3-269.8)	0.1206	-0.74
Tyrosine	78.3 (60.8-95.9)	109.7 (86.9-118.4)	0.1206	-0.81
Uridine	173.5 (129.2-187.8)	169.8 (151.4-199.3)	0.5358	-0.37
Valine	195.9 (155.7-236.9)	222.5 (170.2-234.3)	0.5358	-0.31

*Metabolites that were significantly different after comparison
†Metabolites that trended toward a difference after comparison

Supplementary Table 4 Mean gastrocnemius metabolite concentrations (nmol/g), and comparison between mice on a KD vs CD using a Welch's t-test and Hedge's g effect size measure. For each metabolite, log transformed mean ± standard deviation (SD), p-value (p), and Hedge's g effect size (*g*) are indicated. Metabolites considered significantly different have a $p < 0.05$, and at least a large effect size, $|g| \geq 0.8$. Metabolites trending toward a difference have a $p < 0.1$, and at least a large effect size, $|g| \geq 0.8$

**Metabolite**	**Control, Mean ± SD**	**Ketogenic, Mean ± SD**	***p***	***g***
β-Hydroxybutyrate*	2.2 ± 0.33	2.63 ± 0.17	0.0072	-1.57
Acetone	1.06 ± 0.05	1.11 ± 0.07	0.1119	-0.81
Alanine†	3.16 ± 0.09	3.06 ± 0.12	0.0882	0.87
Aspartate	2.3 ± 0.10	2.24 ± 0.21	0.4734	0.35
Creatine	4.27 ± 0.07	4.24 ± 0.06	0.3611	0.45
Creatine phosphate	3.93 ± 0.06	3.99 ± 0.13	0.2758	-0.55
Dimethyl sulfone	1.85 ± 0.09	1.82 ± 0.07	0.3900	0.42
Formate	2.44 ± 0.08	2.44 ± 0.05	0.8310	-0.10
Fumarate	1.4 ± 0.09	1.42 ± 0.10	0.6840	-0.20
Glucose	3.02 ± 0.10	3.03 ± 0.15	0.8599	-0.09
Glutamate*	2.9 ± 0.06	3.03 ± 0.08	0.0020	-1.80
Glutamine	3.19 ± 0.07	3.23 ± 0.10	0.3269	-0.48
Glycerol	2.79 ± 0.08	2.82 ± 0.05	0.3255	-0.48
Glycine†	3.24 ± 0.12	3.37 ± 0.12	0.0594	-0.97
Isoleucine	1.8 ± 0.06	1.84 ± 0.13	0.4548	-0.37
Leucine	1.87 ± 0.05	1.91 ± 0.06	0.1618	-0.70
Lysine	2.65 ± 0.13	2.56 ± 0.06	0.1123	0.82
NAD+	2.72 ± 0.04	2.73 ± 0.07	0.9730	-0.02
Pyruvate	2.17 ± 0.09	2.14 ± 0.08	0.5066	0.32
Succinate	2.44 ± 0.08	2.47 ± 0.14	0.6242	-0.24
Taurine	4.55 ± 0.06	4.57 ± 0.07	0.4718	-0.35
Tyrosine	1.78 ± 0.18	1.84 ± 0.15	0.4763	-0.35
Valine	2.14 ± 0.05	2.12 ± 0.08	0.4661	0.36

*Metabolites that were significantly different after comparison
†Metabolites that trended toward a difference after comparison

Supplementary Table 5 Comparison of gastrocnemius metabolites (nmol/g) where normality couldn't be approximated using log transformation, using the Wilcoxon-rank sum test and Hedge's g effect size measure. For each metabolite, median and interquartile range (IQR), p-value (*p*), and Hedge's g effect size (*g*) are indicated. Metabolites considered significantly different have a $p < 0.05$, and at least a large effect size, $|g| \geq 0.8$. Metabolites trending toward a difference have a $p < 0.1$, and at least a large effect size, $|g| \geq 0.8$

**Metabolite**	**Control, Median (IQR)**	**Ketogenic, Median (IQR)**	***p***	**ES**
Glutathione	287.1 (277.3-300.2)	310.5 (278.7-376.5)	0.2786	-0.74
Lactate	6032.4 (5819.4-6530.9)	6011.8 (4640.7-6219.3)	0.4418	0.74

Supplementary Table 6 Median hippocampal metabolite concentrations (nmol/g), and comparison between mice on a KD vs CD using the Wilcoxon-rank sum test and Hedge's g effect size measure. For each metabolite, median and interquartile range (IQR), p-value (*p*), and Hedge's g effect size (*g*) are indicated. Metabolites considered significantly different have a $p < 0.05$, and at least a large effect size, $|g| \geq 0.8$. Metabolites trending toward a difference have a p < 0.1, and at least a large effect size, $|g| \geq 0.8$

**Metabolite**	**Control, Median (IQR)**	**Ketogenic, Median (IQR)**	***p***	***g***
β-Hydroxybutyrate*	69.7 (54.9-91.2)	200.1 (131.2-303.7)	0.0093	-1.57
4-Aminobutyrate	1660.7 (1543.8-1856)	2027 (1767.1-2203.4)	0.1206	-0.89
ADP	76.4 (68.9-131.8)	148.8 (102.6-163.8)	0.1206	-0.84
AMP	112.4 (98.7-118.3)	158.9 (130.4-177.6)	0.2319	-0.75
ATP	33.2 (28.8-34.5)	31.4 (26.8-37.6)	0.6943	0.20
Acetate	105.4 (90.9-518.6)	303.7 (144.3-432.9)	0.6943	-0.08
Acetoacetate	14.5 (11.9-15.8)	12.8 (12-16.1)	0.7789	0.35
Adenosine†	136.6 (125.6-140.9)	205.4 (156.2-236.7)	0.0721	-1.12
Alanine	782.9 (685.3-874.3)	878.9 (703.9-976.7)	0.4634	-0.44
Ascorbate	1383.3 (1115.4-1469)	1429.9 (1231.8-1506.1)	0.8665	-0.09
Aspartate	1359.2 (1274-1614.6)	1750.7 (1508.4-1936.9)	0.1206	-0.86
Betaine	112.7 (97.4-121.5)	127.9 (119.2-152.5)	0.1520	-0.85
Choline*	88.2 (75.1-95.2)	113.4 (111.5-117.6)	0.0140	-1.53
Citrate	229 (205.2-282.4)	318.7 (227.5-354.6)	0.1893	-0.57
Creatine	8731.8 (7509.6-9583.1)	10043.8 (9198.3-10662.5)	0.1893	-0.71
Formate	201.6 (193.4-353.5)	291.9 (255-321.5)	0.7789	-0.27
Fumarate*	45.6 (44.1-54)	66.8 (62.5-72.9)	0.0205	-1.46
Glucose	100.8 (51.9-112.6)	92.7 (65.7-122.2)	0.8665	-0.12
Glutamate	7299.3 (6863.6-7923.7)	8876.9 (7825-9667.9)	0.1520	-0.78
Glutamine	3374.8 (3166.1-3996.2)	4632.5 (3667.5-5500.3)	0.2319	-0.82
Glutathione	445.4 (388.2-490.9)	568.8 (475.5-602.3)	0.1206	-0.92
Glycerol	319.5 (302.9-379.1)	401 (375.9-415.1)	0.1520	-0.77
Glycine*	819.3 (766.5-966.3)	1055.5 (985.5-1113.6)	0.0093	-1.61
Inosine†	56.4 (53.5-62.3)	68.6 (63.1-74.3)	0.0721	-0.88
Isoleucine	40 (38.1-45.5)	38.9 (31.7-47.5)	0.7789	0.21
Lactate	9419.9 (7562.6-10928.8)	10247.5 (9182.8-10752.9)	0.6126	-0.44
Leucine	48.8 (37.5-56.4)	50.7 (43.5-53)	>0.9999	-0.18
Lysine	94.5 (93.7-102.9)	101.2 (95-150.4)	0.5358	-0.34
Methionine	72.7 (62.8-76.2)	74.8 (69.6-76.7)	0.6943	0.09
Myo-inositol	4001.3 (3507-4729.6)	4745.1 (4308.1-5308.7)	0.1520	-0.73

NAD+	100.8 (95.2-112.6)	124.7 (97.6-137.7)	0.3357	-0.65
NADH	28 (20.3-31)	23.5 (16.1-31.4)	0.9551	0.13
Ornithine*	59.2 (53.6-66)	78.3 (72.5-89.4)	0.0205	-1.39
Pantothenate*	32.5 (28.6-45.4)	51.1 (47.2-56.8)	0.0093	-1.62
Phenylalanine	40.9 (37.6-50.5)	46 (39.3-52.1)	0.9551	0.01
Pyruvate†	43.6 (42.6-58.9)	58 (53.8-83)	0.0939	-1.07
Succinate	237.3 (181.7-254.2)	253.7 (225.9-278.8)	0.3357	-0.56
Taurine	8296.9 (6859.1-9067.5)	9385 (8653.9-10106.1)	0.2319	-0.68
Threonine	195.1 (170.4-273.5)	196.5 (186-232.9)	0.9551	0.19
Tryptophan	61.8 (55.8-62.6)	64.1 (58-66.4)	0.4634	0.16
Valine	78 (70-91.9)	77.7 (67.7-89.4)	0.8665	0.07

*Metabolites that were significantly different after comparison
†Metabolites that trended toward a difference after comparison

Supplementary Table 7 Median cortex metabolite concentrations (nmol/g), and comparison between mice on a KD vs CD using the Wilcoxon-rank sum test and Hedge's g effect size measure. For each metabolite, median and interquartile range (IQR), p-value (*p*), and Hedge's g effect size (*g*) are indicated. Metabolites considered significantly different have a $p < 0.05$, and at least a large effect size, $|g| \geq 0.8$. Metabolites trending toward a difference have a $p < 0.1$, and at least a large effect size, $|g| \geq 0.8$

Metabolite	Control, Median (IQR)	Ketogenic, Median (IQR)	*p*	*g*
β-Hydroxybutyrate*	91.6 (48.2-114)	203.8 (156.2-311.8)	0.0104	-1.50
4-Aminobutyrate	2172.3 (2027-2264.9)	1977.1 (1740.8-2230.9)	0.3282	0.61
ADP	212.6 (196.6-266.6)	205.9 (192-235.6)	0.5054	0.42
AMP	178.6 (137.9-194.4)	180.9 (149.3-186.9)	0.9591	0.12
ATP	35 (31.5-37.1)	34.5 (29.5-36.6)	0.8785	-0.14
Acetoacetate	10.1 (6.3-10.8)	10.4 (9.8-11.2)	0.4418	-0.34
Adenosine	228.7 (174.1-251.9)	231.4 (183.5-236.1)	0.7984	0.12
Alanine	733.7 (691.1-817.2)	701.5 (642.5-740.2)	0.2345	0.79
Ascorbate	1141.1 (1060.6-1167.2)	867.7 (804.5-1155.1)	0.2345	0.83
Aspartate	2706.3 (2534.4-2971)	2620.3 (2374.4-2818.7)	0.2786	0.66
Betaine*	155.6 (140.3-172.5)	123.5 (116.3-129.5)	0.0030	1.57
Choline	88.8 (83.5-93.4)	82.5 (73.5-88.1)	0.3823	0.40
Creatine	9486.3 (8898.5-10256)	9358 (7315.1-9893.1)	0.3823	0.78
Formate	225.6 (206.4-242.6)	211 (202.6-242.6)	0.6454	0.38
Fumarate*	54.6 (52-57.9)	46.7 (44.5-49.1)	0.0281	1.16
Glucose	52.6 (44.8-53.4)	61.8 (48-69.8)	0.1605	-0.73
Glutamate	11046.2 (10103.1-11968.2)	10855.6 (9176.7-11409.2)	0.2345	0.64
Glutamine	5210.6 (4672-5837.7)	4722.1 (4485.2-5911.2)	0.6454	0.08
Glutathione	517.7 (444.7-578.2)	429.6 (397.8-598.1)	0.6454	0.11
Glycerol	296.1 (268.4-317.1)	299.4 (254.1-318.9)	0.9591	0.03
Glycine	915.8 (881.7-980.7)	989.4 (885-1014.4)	0.8785	0.16
Inosine†	52.3 (50.1-73)	75.2 (63-80.1)	0.0650	-0.99
Isoleucine	37.1 (34-41.6)	35.1 (33.5-36.3)	0.2786	0.61
Lactate*	11351.6 (9852.1-12578)	9429.1 (8223.6-10107.6)	0.0281	1.23
Leucine	54.7 (51.6-56.9)	51.8 (46.9-53.9)	0.1605	0.55
Lysine*	153.4 (147.6-170.8)	126.1 (119.3-130.4)	0.0047	1.64
Methionine	79 (71.5-88.9)	79.7 (68.7-87.5)	0.8785	0.30
Myo-inositol	4715.5 (4500.8-5444.6)	4249.7 (3773.6-4966.3)	0.2786	0.71
NAD+	251.6 (226.2-299.3)	249.4 (241.5-265.3)	0.7984	0.42
NADH	47.7 (45.3-52.7)	41.9 (37.4-49)	0.3282	0.43

Ornithine	88.5 (83.7-91.6)	77.3 (73.9-80.1)	0.1304	0.71
Pantothenate	50.2 (46.1-52)	53.5 (42.2-58.2)	0.5737	-0.27
Phenylalanine	47.6 (42.5-52.7)	47.4 (37.8-56)	0.8785	0.19
Pyruvate	54.6 (40.9-65.1)	44 (40.7-48.6)	0.3282	0.63
Succinate	403.5 (362.9-439.6)	434.5 (330.7-450.2)	0.9591	-0.05
Taurine	10751.4 (10184.6-11760.2)	10162.7 (8655.3-11560.4)	0.3823	0.70
Threonine	178.8 (148.8-247.4)	151.4 (146.8-179.1)	0.3823	0.68
Tryptophan	32.6 (23.1-34.7)	27.9 (25.2-33.7)	0.5992	0.34
Tyrosine	57.2 (49.1-64)	57.5 (50.4-72.8)	0.7209	-0.25
Uridine	58.7 (48.1-64.7)	62.7 (42.6-71.3)	0.7984	-0.24
Valine	88.2 (84-108.2)	80.7 (60.6-88.3)	0.1049	0.98

*Metabolites that were significantly different after comparison
†Metabolites that trended toward a difference after comparison
